# Awareness of tobacco control policies and anti-tobacco attitudes and behaviors among school personnel

**DOI:** 10.18332/tid/149926

**Published:** 2022-06-09

**Authors:** Yin-Liang Tan, Zi-Yue Chen, Ya-Ping He, Gang Xu, Zhi-Ping Yu, Jing-Fen Zhu

**Affiliations:** 1 School of Public Health, Shanghai Jiao Tong University School of Medicine, Shanghai, China; 2 Department of Nutrition and Dietetics, University of North Florida, Jacksonville, United States

**Keywords:** adolescent health, smoke-free policy, school personnel, smoking reduction, school health

## Abstract

**INTRODUCTION:**

Adolescent smoking is a serious public health concern, and the role of personnel in reducing students’tobacco use has been proven. Anti-tobacco policies are strong factors for tobacco control but most are newly implemented in China. This study aimed to examine the awareness of anti-tobacco policies among school personnel in a southern city of China, and assess its influence on personnel’s anti-tobacco attitudes and behaviors towards students.

**METHODS:**

An online cross-sectional study was conducted between September 2017 and January 2018 in schools of Shanghai, China. A total of 3194 subjects from 33 schools were selected by a two-stage stratified cluster randomized sampling design. Prevalence of anti-tobacco policy awareness is presented. Crude (ORs) and adjusted odds ratios (AORs) and their 95% confidence intervals (CIs) were estimated to assess the association between policy awareness and anti-tobacco attitudes or behaviors.

**RESULTS:**

In all, 22.4% of surveyed participants knew four or five polices presented in the survey and 13.0% of personnel knew none of these policies. Most of the participants fully support prohibiting indoor (94.6%) and outdoor (86.3%) smoking in public places, bans on tobacco advertising (90.9%), and printing warning pictures on cigarette boxes (89.5%). Less than half of the personnel had taken action to stop students from smoking (45.7%), encourage students to quit smoking (42.4%) or participated in relevant educational activities held by schools (37.4%) in the previous year. The school personnel’s anti-tobacco attitudes (AOR=1.28; 95% CI: 1.21–1.36) and behaviors (AOR=1.10; 95% CI: 1.03–1.17) were strengthened with increasing level of policy awareness.

**CONCLUSIONS:**

The involvement of school personnel can be an important part of intervention to improve anti-tobacco campaigns on campus. The study calls for the implementation of projects or activities to improve anti-tobacco policy awareness in the school environment as part of school tobacco control strategy.

## INTRODUCTION

Tobacco use in early life has been shown to negatively impact physical (e.g. pulmonary and cardiovascular diseases)^1^ or psychological (e.g. depression and anxiety disorders)^2^ health both immediately and in the future^3^. It also increases the likelihood of smoking later in adulthood^3^. In China, smoking prevalence remains high, especially in schools. For example, the 2019 Middle School Students Tobacco Survey conducted in China revealed that 24.5% of senior high school students have tried smoking, and the proportion was even higher in vocational high school students (30.3%)^4^. In addition, electronic cigarettes have become increasingly popular among teens. It was also reported that the proportion of Chinese junior middle school students that knew of e-cigarettes increased from 45.0% in 2014 to 69.9% in 2019^4^. Despite of the high smoking prevalence, according to the WHO report, China was among 59 countries that have not fully implemented the MPOWER^5^ policy package: Monitoring tobacco use; Protecting people from the tobacco; Offering help to quit tobacco; Warning about the dangers; Enforcing bans on advertising, promotion and sponsorship; and Raising tobacco taxes.

School personnel could play an important role in adolescent smoking^6,7^. On the one hand, smoking status of school personnel influences students’ smoking. A study focusing on social-cognitive factors among adolescent smoking status showed that teachers’ norms on tobacco use was a significant contributor^8^. High visibility of teacher’s smoking was related to an increasing smoking rate among students^7^. On the other hand, school personnel could help students stay away from tobacco. In one study, positive relationships were observed between teachers’ anti-tobacco norms and smoke-free school policies perceived by students^9^. In another study, 70% of the junior middle school personnel in Taiwan have advised their students to quit smoking, which was associated with a decrease in students’ tobacco use^10^. Clearly, school personnel’s anti-tobacco attitudes and behaviors are closely associated with adolescent tobacco use. When it comes to the adolescent smoking control on campus, improving the tobacco control attitudes and behaviors among school personnel can be beneficial.

Studies have reported that school personnel’s attitudes and behaviors were positively associated with their anti-tobacco policy awareness^11,12^. Raising school personnel’s awareness of anti-smoking policies might be important to form their anti-smoking attitudes and behaviors and later reduce smoking among their students. To date, many policies have been proposed and implemented around the world in response to the globalization of tobacco epidemic. WHO MPOWER is a policy package developed by WHO Framework Convention on Tobacco Control (FCTC) to reverse the tobacco epidemic^13^. From 2007 to 2014, MPOWER policy was adopted in 88 countries, contributing to the decreased number of smoking-attributable deaths by almost 22 million^14^. In alignment with initiatives of FCTC, the Chinese government amended Advertisement Law (AL) of People’s Republic of China in 2015 to regulate tobacco advertising in all mass media and public places^15^. Published in 2016, the national action plan Healthy China 2030 aims to reduce smoking rate of people aged ≥15 years to 20% by the year of 2030. To meet this goal, several cities have established or amended local tobacco control laws and regulations. In Shanghai, the Regulations of Shanghai Municipality on Smoking Control in Public Places was revised to prohibit smoking in all indoor workplaces and in many public outdoor areas. Since the implementation in 2017, smoking rate in public places of Shanghai had dropped to 12.8% by 2020^16^.

Though many anti-tobacco policies are in place, the awareness of those policies among the public is alarmingly low in many countries including China. In Nigeria, about one-third of young people had never heard of any tobacco control law, and 48.1% did not known about the WHO FCTC^17,18^. Data from a survey in Pakistan has indicated that 46.1% of the students had never noticed smoke-free policies around them^19^. The relationship between knowledge-attitude-belief and practice (KABP)^20^ has been widely applied in research on health behavior and decision-making theories^21,22^, and was important in the implications for tobacco control^23^. However, to the best of our knowledge, few published original studies have examined the awareness of anti-tobacco policies as part of the knowledge, which is actually a very simple but valuable perception, rather than exploring its relationship with people’s attitudes and behaviors, especially among school personnel groups. In the current study, we aimed to assess the awareness of anti-tobacco policies among school personnel and evaluate its association with their tobacco control attitudes and behaviors towards students. The findings will provide important evidence for the government to develop school tobacco control strategies with the improvement of school personnel’s role.

## METHODS

### Study design and participants

This cross-sectional study with a two-stage stratified cluster randomized sampling design was carried out between September 2017 and January 2018 in Shanghai, China. In the first stage, we conducted the selection of districts randomly from all the 16 districts according to location: Huangpu District and Putuo District as samples for urban areas and Minhang District, Jiading District for rural areas. The second stage involved the selection of schools based on proportion-to-size sampling approach (17 for junior middle schools, 10 for senior high schools and 6 for vocational high schools). Finally, all the 3311 school personnel working in the 33 schools were included, covering administrators, teachers and other staff.

The questionnaires were filled in anonymously on a platform for questionnaire surveys (https://www.wjx.cn/). The survey was a modified design of the Global School Personnel Survey (GSPS) initiated by WHO^24^ and ‘questionnaires for monitoring smoking related behaviors of key population’ from the Chinese Center for Disease Control and Prevention.

### Measures

*Awareness of tobacco control policies*

The awareness of tobacco control policies was assessed by asking: ‘Which of the following tobacco control policies have you heard of?’. The options shown in our questionnaire included: WHO FCTC, MPOWER, Regulations of Shanghai Municipality on Smoking Control in Public Places, Advertisement Law (AL) of People’s Republic of China, and Healthy China 2030. Based on their answers, we categorized the participants into three groups: those who knew none of the policies, those who knew 1–3 types, and those who knew 4 or 5 types.

*Attitudes towards tobacco control*

To estimate their attitudes towards tobacco control measures which had already been put into practice, participants were asked: ‘Do you support the 'comprehensive bans on tobacco advertising'?’, ‘What is your attitude towards prohibiting smoking in indoor public places (such as schools, shops, restaurants, cinemas, airports, etc.)?’, ‘What is your attitude towards prohibiting outdoor smoking in public places (such as parks/squares/open-air sports venues/bus stations, etc.)?’, and ‘Do you support printing warning pictures of tobacco-related diseases on cigarette boxes in China?’. Answers were set on a four-point Likert-type scale: those who fully support, partially support, do not care, or not support any measure. Based on their answers, we then categorized the participants into two groups: the ‘Totally support’ group including those who chose ‘Fully support’ for all four questions and ‘Others’ group covering the rest of participants.

*Behaviors related to tobacco control*

Participants were asked whether they adopted the following behaviors and activities in the past one year: 1) prevented students from smoking, 2) advised students to quit smoking, and 3) participated in any educational activities related to tobacco control for students held by schools. Personnel that chose ‘Yes’ for all three questions were classified as ‘Totally participants’ and the rest were classified as ‘Others’.

### Other variables

Potential confounders were district (urban or rural), school type (junior middle school, senior high school or vocational high school), age (20–29, 30–39, 40–49, 50–65 years), sex (male or female), working duration (<10 years or ≥10 years), educational level (college and below, Bachelor’s and above), and position (teachers, administrators or other staff). Personnel were also classified by their smoking status: ‘never smoker’, ‘former smoker’ and ‘current smoker’. ‘Current smoker’ refers to the personnel who smoke currently, including daily smokers and those who smoked occasionally. Participants who had never smoked were referred to as ‘never smoker’, while those with smoking experience but had quit smoking successfully at the moment of the survey were referred to as ‘former smoker’. Also, personnel’s involvement in tobacco control training was measured by asking: ‘Have you ever received any relevant training to prevent teenage smoking?’

### Statistical analysis

All data analysis was performed using SPSS 22.0 (SPSS Statistic Inc., Chicago, IL, USA) complex sampling analysis procedure to provide weighted results that accounted for the complex sample design after excluding missing data. A chi-squared test was used to compare the differences in categorical variables between groups. Unadjusted (ORs) and adjusted odds ratios (AORs) and 95% confidence intervals (95% CI) were calculated using multiple logistic regression analysis to explore the associations between the independent variable (awareness level of tobacco control policy) and dependent variables (attitude and behavior of tobacco control). Adjusted odds ratios were controlled for district, school type, age, sex, work duration, education level, position in school, training of tobacco control and smoking status. Awareness of tobacco control policies was treated as both ordinal variables and continuous variable in models. A threshold for statistical significance was set at a two-sided p<0.05 level.

## RESULTS

### Sample characteristics

In total, 3311 school personnel from 33 schools participated in the survey. The final sample size was 3194 (response rate of 96.47%), after removing those not passing the data quality check (n=117), e.g. finished the questionnaire too quickly (<300 s). As shown in Table 1, 60.7% of the participants were from rural areas, 65.2% were aged 30–49 years, 73.3% were females and 80.6% were teachers. Sixty-eight percent had worked for over 10 years, and 91.6% had a Bachelor’s degree or above. Regarding the school type, 58.2% worked in junior middle schools, 27.0% in senior high schools, and 14.8% in vocational high schools. Among all the participants, 7.4% were currently smoking and 84.1% had never received any training on teenage smoking prevention.

**Table 1:** Demographic characteristics, policy awareness, tobacco control attitude and behavior of school personnel, Shanghai, China 2017–2018 (N=3194)

*Variables*	*Weighted*		*Unweighted*	
	*%*	*95% CI*	*Population*	*n*
**District**				
Urban	39.3	37.6–41.0	33979	1281
Rural	60.7	59.0–62.4	52576	1913
**School type**				
Junior middle school	58.2	56.5–59.9	50394	1719
Senior high school	27.0	25.4–28.7	23411	681
Vocational high school	14.8	13.8–15.8	12750	794
**Age** (years)				
20–29	16.9	15.6–18.2	14581	518
30–39	30.3	28.7–32.0	26261	961
40–49	34.9	33.3–36.6	30239	1127
50–65	17.9	16.6–19.3	15475	588
**Sex**				
Male	26.7	25.2–28.2	23082	887
Female	73.3	71.8–74.8	63474	2307
**Work duration** (years)				
<10	32.0	30.4–33.7	27693	1028
≥10	68.0	66.3–69.6	58862	2166
**Education level**				
College and below	8.4	7.5–9.4	7283	319
Bachelor’s and above	91.6	90.6–92.5	79272	2875
**Position**				
Teacher	80.6	79.2–81.9	69775	2521
Administrator	7.3	6.4–8.2	6279	241
Other staff	12.1	11.1–13.3	10501	432
**Training of tobacco control**				
No	84.1	82.8–85.3	72781	2677
Yes	15.9	14.7–17.2	13775	517
**Smoking status**				
Never	88.8	87.7–89.9	76884	2805
Former	3.8	3.2–4.5	3299	128
Current	7.4	6.5–8.3	6373	261
**Policy awareness ^a^**				
None	13.0	11.8–14.2	11216	427
1–3 types	64.6	62.9–66.3	55952	2039
4–5 types	22.4	21.0–23.9	19387	728
**Support attitude to policy^b^**				
Others	24.9	23.4–26.4	21515	827
Totally support	75.1	73.6–76.6	65041	2367
**Tobacco control behavior in school^c^**				
Others	82.3	80.9–83.6	71221	2581
Totally participants	17.7	16.4–19.1	15334	613

Respondents not passing the data quality check (n=117) were not present during survey.

a Policy awareness: the number of anti-tobacco policies that the participant having heard of among all the five policies listed in the survey.

b Attitudes towards four kinds of anti-tobacco measures listed in the survey. Totally support: referring to the participants who fully support all the measures.

c The situation of adopting tobacco control behaviors in the past year. Totally participants: referring to the participants who had adopted all three kinds of behaviors listed in the questionnaire.

### Tobacco control policy awareness, attitudes and behaviors among personnel

Regarding the tobacco control policies, the proportion of school personnel having heard of the WHO FCTC (49.7%), MPOWER (29.6%) and AL of People’s Republic of China (49.8%) were all lower than 50%, except for Regulations of Shanghai Municipality on Smoking Control in Public Places (68.3%). In addition, the proportion of participants knowing Healthy China 2030 was the lowest (25.4%). Among the five different types, 22.4% of the participants were aware of 4–5 types and 13.0% knew none of the policies. As for personnel’s attitudes towards four types of anti-tobacco measures, the rate of fully supportive was higher than 85% in every type, while the proportion of participants who fully supported all four measures (totally support) was 75.1%. Regarding anti-tobacco behaviors in school, less than half of the school personnel tried to prevent students from smoking (45.7%) or encourage them to stop smoking (42.4%), yet 37.4% had participated in smoking-related educational activities on campus in the previous year. The proportion of participants endorsing all three kinds of tobacco control behaviors in school was 17.7%.

### Relationship between policy awareness and tobacco control attitudes and behaviors

The awareness of anti-tobacco policies was positively associated with supportive attitudes towards tobacco control (Table 2), especially for outdoor smoking banning and anti-tobacco warning pictures (p<0.05 in every type of policy). For example, rate of fully supporting anti-tobacco warning pictures was higher in participants knowing FCTC (94.0%) than those having not heard of it (85.1%). Besides, higher awareness also showed significantly higher chance of anti-tobacco behaviors in schools, with exception of the relationship between Regulations of Smoking Control in Public Place and advising students to quit smoking/preventing students from smoking as well as the difference between Healthy China 2030 and advising students to quit smoking.

**Table 2:** Supportive attitudes to anti-tobacco measurements and tobacco control behaviors stratified by awareness of different policies among the total sample [% (95% CI)], Shanghai, China 2017–2018 (N=3194)

	*Total*	*FCTC ^c^*			*MPOWER ^d^*			*Healthy China 2030*			*Regulations of smoking control in public place*			*Advertisement law*		
		*No*	*Yes*	*p*	*No*	*Yes*	*p*	*No*	*Yes*	*p*	*No*	*Yes*	*p*	*No*	*Yes*	*p*
**Indoor banning^a^**																
Fully support	94.6 (93.7–95.3)	94.0 (92.7–95.0)	95.1 (94.0–96.1)	0.140	94.5 (93.5–95.4)	94.7 (93.2–95.9)	0.802	94.3 (93.3–95.2)	95.2 (93.6–96.5)	0.313	91.1 (89.2–92.7)	96.2 (95.3–96.9)	<0.001	93.0 (91.7–94.2)	96.1 (95.1–96.9)	<0.001
Others	5.4 (4.7–6.3)	6.0 (5.0–7.3)	4.9 (3.9–6.0)		5.5 (4.6–6.5)	5.3 (4.1–6.8)		5.7 (4.8–6.7)	4.8 (3.5–6.4)		8.9 (7.3–10.8)	3.8 (3.1–4.7)		7.0 (5.8–8.3)	3.9 (3.1–4.9)	
**Outdoor banning^a^**																
Fully support	86.3 (85.1–87.5)	84.5 (82.6–86.2)	88.2 (86.5–89.7)	0.002	85.2 (83.7–86.6)	88.9 (86.8–90.7)	0.005	85.2 (83.7–86.5)	89.7 (87.5–91.6)	0.001	83.2 (80.8–85.4)	87.8 (86.3–89.1)	0.001	84.5 (82.6–86.2)	88.1 (86.5–89.6)	0.003
Others	13.7 (12.5–14.9)	15.5 (13.8–17.4)	11.8 (10.3–13.5)		14.8 (13.4–16.3)	11.1 (9.3–13.2)		14.8 (13.5–16.3)	10.3 (8.4–12.5)		16.8 (14.6–19.2)	12.2 (10.9–13.7)		15.5 (13.8–17.4)	11.9 (10.4–13.5)	
**Advertising banning^a^**																
Fully support	90.9 (89.9–91.9)	89.8 (88.3–91.2)	92.0 (90.5–93.2)	0.038	90.6 (89.3–91.7)	91.7 (89.8–93.3)	0.288	89.8 (88.5–91.05)	94.1 (92.2–95.5)	<0.001	87.7 (85.5–89.6)	92.4 (91.2–93.5)	<0.001	88.9 (87.3–90.4)	92.9 (91.5–94.1)	<0.001
Others	9.1 (8.1–10.1)	10.2 (8.8–11.8)	8.0 (6.8–9.5)		9.4 (8.3–10.7)	8.3 (6.7–10.2)		10.2 (9.0–11.5)	5.9 (4.5–7.8)		12.3 (10.4–14.5)	7.6 (6.5–8.8)		11.1 (9.6–12.7)	7.1 (5.9–8.5)	
**Warning picture^a^**																
Fully support	89.5 (88.4–90.5)	85.1 (83.2–86.7)	94.0 (92.7–95.1)	<0.001	88.1 (86.7–89.4)	92.9 (91.1–94.4)	<0.001	87.9 (86.5–89.2)	94.1 (92.3–95.6)	<0.001	86.0 (83.7–88.0)	91.1 (89.9–92.3)	<0.001	86.7 (85.0–88.3)	92.3 (90.8–93.5)	<0.001
Others	10.5 (9.5–11.6)	14.9 (13.3–16.8)	6.0 (4.9–7.3)		11.9 (10.6–13.3)	7.1 (5.6–8.9)		12.1 (10.8–13.5)	5.9 (4.4–7.7)		14.0 (12.0–16.3)	8.9 (7.7–10.1)		13.3 (11.7–15.0)	7.7 (6.5–9.2)	
**Take part in educational activities on campus^b^**																
No	62.6 (60.9–64.3)	69.2 (66.9–71.4)	56.0 (53.5–58.5)	<0.001	66.2 (64.1–68.1)	54.3 (51.1–57.5)	<0.001	66.2 (64.3–68.1)	52.1 (48.7–55.6)	<0.001	68.9 (66.0–71.8)	59.7 (57.6–61.8)	<0.001	67.9 (65.5–70.2)	57.3 (54.9–59.8)	<0.001
Yes	37.4 (35.7–39.1)	30.8 (28.6–33.1)	44.0 (41.5–46.5)		33.8 (31.9–35.9)	45.7 (42.5–48.9)		33.8 (31.9–35.7)	47.9 (44.4–51.3)		31.1 (28.2–34.0)	40.3 (38.2–42.4)		32.1 (29.8–34.5)	42.7 (40.2–45.1)	
**Prevent students from smoking^b^**																
No	54.3 (52.6–56.0)	57.8 (55.3–60.2)	50.8 (48.3–53.3)	<0.001	56.9 (54.8–58.9)	48.2 (45.0–51.4)	<0.001	55.8 (53.8–57.8)	50.0 (46.5–53.4)	0.005	56.3 (53.2–59.4)	53.4 (51.2–55.5)	0.121	56.8 (54.4–59.3)	51.8 (49.3–54.2)	0.004
Yes	45.7 (44.0–47.4)	42.2 (39.8–44.7)	49.2 (46.7–51.7)		43.1 (41.1–45.2)	51.8 (48.6–55.0)		44.2 (42.2–46.2)	50.0 (46.6–53.5)		43.7 (40.6–46.8)	46.6 (44.5–48.8)		43.2 (40.7–45.6)	48.2 (45.8–50.7)	
**Advise students to quit smoking^b^**																
No	57.6 (55.8–59.3)	61.0 (58.6–63.3)	54.1 (51.6–56.6)	<0.001	59.8 (57.7–61.8)	52.2 (49.0–55.4)	<0.001	58.5 (56.5–60.5)	54.6 (51.2–58.1)	0.053	58.6 (55.6–61.7)	57.0 (54.9–59.1)	0.396	60.2 (57.8–62.6)	54.9 (52.4–57.3)	0.002
Yes	42.4 (40.7–44.2)	39.0 (36.7–41.4)	45.9 (43.4–48.4)		40.2 (38.2–42.3)	47.8 (44.6–51.0)		41.5 (39.5–43.5)	45.4 (41.9–48.8)		41.4 (38.3–44.4)	43.0 (40.9–45.1)		39.8 (37.4–42.2)	45.1 (42.7–47.6)	
Total		50.3 (48.6–52.1)	49.7 (47.9–51.4)		70.4 (68.7–71.9)	29.6 (28.1–31.3)		74.6 (73.0–76.1)	25.4 (23.9–27.0)		31.7 (30.1–33.4)	68.3 (66.6–69.9)		50.2 (48.4–51.9)	49.8 (48.1–51.6)	

a Attitudes towards four kinds of tobacco control measures. Others: including the school personnel who partly support, do not care or not support the tobacco control measure.

b Activities and behaviors that school personnel adopted in the past year.

c WHO Framework Convention on Tobacco Control.

d Monitoring tobacco use; Protecting people from the tobacco smoke; Offering help to quit tobacco; Warning the public about the dangers; Enforcing bans on advertising, promotion and sponsorship; and Raising tobacco taxes policy package.

### Policy awareness, tobacco control attitudes and behaviors in school personnel with different characteristics

Table 3 shows the distributions stratified by various influencing factors including sociodemographic factors, work duration, position, smoking status and training experience. Personnel in urban areas (compared to rural area, p<0.01) or with longer work duration (≥10 years, compared to <10 years, p<0.001) were related to increased policy awareness, anti-tobacco attitudes and behaviors. Participants who received tobacco control training knew more types of anti-tobacco policies and had taken more actions to prevent/stop smoking among students compared to those without training experiences (p<0.001). Besides, school administrators were more likely to take anti-tobacco behaviors (28.8%) than school teachers (17.6%) or other staff (12.1%) (p<0.001). Majority of school administrators (90.1%) knew one to five policies given, higher than teachers (87.3%) and other staff (83.9%). Current smokers (29.3%) were less likely to fully support all four anti-tobacco measures than never (79.5%) or former (62.4%) smokers.

**Table 3:** Policy awareness, support attitude to policy and tobacco control behaviors in school across different characteristic of school personnel among the total sample [% (95% CI)], Shanghai, China 2017–2018 (N=3194)

	*Policy awareness ^a^*				*Attitudes towards policy ^b^*			*Tobacco control behavior in school ^c^*		
	*None*	*1–3 types*	*4–5 types*	*p*	*Others*	*Totally support*	*p*	*Others*	*Totally participants*	*p*
**District**										
Urban	10.2 (8.7–12.0)	65.9 (63.2–68.4)	23.9 (21.6–26.4)	0.001	22.0 (19.8–24.3)	78.0 (75.7–80.2)	0.002	79.3 (77.0–81.4)	20.7 (18.6–23.0)	<0.001
Rural	14.7 (13.2–16.4)	63.9 (61.7–66.0)	21.4 (19.6–23.3)		26.7 (24.8–28.8)	73.3 (71.2–75.2)		84.2 (82.5–85.8)	15.8 (14.2–17.5)	
**School type**										
Junior middle school	12.2 (10.8–13.8)	64.9 (62.6–67.1)	22.9 (21.0–24.9)	0.006	2121.6 (19.7–23.6)	78.4 (76.4–80.3)	<0.001	85.0 (83.3–86.6)	15.0 (13.4–16.7)	<0.001
Senior high school	12.6 (10.4–15.3)	67.6 (64.0–70.9)	19.8 (17.0–22.9)		26.6 (23.4–30.0)	76.6 (65.4–62.0)		83.4 (80.5–86.0)	19.5 (30.5–27.4)	
Vocational high school	16.5 (14.1–19.2)	58.4 (55.0–61.8)	25.1 (22.2–28.1)		34.6 (31.4–38.0)	65.4 (62.0–68.6)		69.5 (66.3–72.6)	30.5 (27.4–33.7)	
**Sex**										
Male	13.5 (11.4–15.9)	62.3 (59.0–65.5)	24.2 (21.4–27.2)	0.229	41.7 (38.4–45.0)	58.3 (55.0–61.6)	<0.001	80.5 (77.7–83.0)	19.5 (17.0–22.3)	0.104
Female	12.8 (11.4–14.2)	65.5 (63.5–67.4)	21.7 (20.1–23.5)		18.7 (17.2–20.4)	81.3 (79.6–82.8)		82.9 (81.4–84.4)	17.1 (15.6–18.6)	
**Work duration** (years)										
<10	15.4 (13.3–17.8)	65.7 (62.7–68.6)	18.9 (16.6–21.4)	<0.001	29.7 (27.0–32.6)	70.3 (67.4–73.0)	<0.001	87.9 (85.8–89.7)	12.1 (10.3–14.2)	<0.001
≥10	11.8 (10.5–13.2)	64.1 (62.1–66.2)	24.1 (22.3–25.9)		22.6 (20.8–24.4)	77.4 (75.6–79.2)		79.7 (77.9–81.3)	20.3 (18.7–22.1)	
**Education level**										
College and below	17.7 (13.8–22.5)	48.0 (42.3–53.6)	34.3 (29.2–39.8)	<0.001	41.8 (36.3–47.4)	58.2 (52.6–63.7)	<0.001	84.8 (80.2–88.5)	15.2 (11.5–19.8)	0.245
Bachelor’s and above	12.5 (11.4–13.8)	66.2 (64.4–67.9)	21.3 (19.8–22.9)		23.3 (21.8–24.9)	76.7 (75.1–78.2)		82.1 (80.6–83.4)	17.9 (16.6–19.4)	
**Position**										
Teachers	12.8 (11.5–14.1)	66.5 (64.6–68.3)	20.8 (19.2–22.4)	<0.001	23.3 (21.7–25.0)	76.7 (75.0–78.3)	<0.001	82.4 (80.9–83.9)	17.6 (16.1–19.1)	<0.001
Administrator	9.9 (6.6–14.6)	64.0 (57.6–69.9)	26.1 (20.8–32.2)		25.3 (20.1–31.2)	74.7 (68.8–79.9)		71.2 (65.1–76.6)	28.8 (23.4–34.9)	
Other staff	16.1 (12.9–20.0)	52.9 (48.0–57.7)	31.0 (26.7–35.7)		35.0 (30.6–39.7)	65.0 (60.3–69.4)		87.9 (84.4–90.7)	12.1 (9.3–15.6)	
**Smoking status**										
Never smoker	12.9 (11.7–14.2)	65.2 (63.3–66.9)	21.9 (20.4–23.5)	0.444	20.5 (19.0–22.1)	79.5 (77.9–81.0)	<0.001	82.5 (81.0–83.8)	17.5 (16.2–19.0)	0.664
Former smoker	12.0 (7.4–18.7)	61.7 (52.9–69.8)	26.3 (19.4–34.7)		37.6 (29.5–46.5)	62.4 (53.5–70.5)		79.5 (71.4–85.7)	20.5 (14.3–28.6)	
Current smoker	13.7 (10.1–18.4)	60.4 (54.2–66.3)	25.9 (20.9–31.6)		70.7 (64.7–76.0)	29.3 (24.0–35.3)		81.6 (76.4–85.9)	18.4 (14.1–23.6)	
**Training of tobacco control**										
No	14.4 (13.1–15.7)	66.1 (64.2–67.8)	19.6 (18.1–21.1)	<0.001	24.9 (23.3–26.6)	75.1 (73.4–76.7)	0.825	86.9 (85.5–88.1)	13.1 (11.9–14.5)	<0.001
Yes	5.6 (3.9–7.9)	57.2 (52.8–61.5)	37.2 (33.1–41.6)		24.5 (20.9–28.4)	75.5 (71.6–79.1)		58.1 (53.7–62.3)	41.9 (37.7–46.3)	

a Policy awareness: the number of anti-tobacco policies that the participant had heard of among all the five policies listed in the survey.

b Attitudes towards four kinds of anti-tobacco measures listed in the survey. Totally support: referring to the participants who fully support all the measures.

c The situation of adopting tobacco control behaviors in the past year. Totally participants: referring to the participants who had adopted all three kinds of behaviors listed in the questionnaire.

### Influence of policy awareness on tobacco control attitudes and behaviors

Table 4 shows the association between policy awareness and supportive attitudes and tobacco control behaviors among school personnel (p<0.001). After adjusting for covariates, compared with personnel who knew none of the policies, those who knew 1–3 types or 4–5 types were 1.74 (95% CI: 1.36–2.21) or 3.54 (95% CI: 2.60–4.82) times more likely to have supportive attitudes. The occurrence of tobacco control behaviors in those who knew 4–5 types of policies were 1.76 (95% CI: 1.23–2.51) times those who did not know any of the policies. When treated as a continuous variable, the policy awareness was still positively associated with anti-tobacco attitudes (AOR=1.28; 95% CI: 1.21–1.36) and behaviors (AOR=1.10; 95% CI: 1.03–1.17).

**Table 4:** Relationship between policy awareness and tobacco control attitude/behavior in school among school personnel, Shanghai, China 2017–2018 (N=3194)

	*Attitude towards policy*				*Tobacco control behavior in school*			
	*OR (95% CI)*	*p*	*AOR (95% CI)*	*p*	*OR (95% CI)*	*p*	*AOR (95% CI)*	*p*
**Policy awareness**								
None (Ref.)	1		1		1		1	
1–3 types	1.75 (1.40–2.18)	<0.001	1.74 (1.36–2.21)	<0.001	1.42 (1.04–1.93)	0.026	1.22 (0.88–1.68)	0.234
4–5 types	2.98 (2.26–3.93)	<0.001	3.54 (2.60–4.82)	<0.001	2.46 (1.77–3.43)	<0.001	1.76 (1.23–2.51)	0.002
p for trend		<0.001		<0.001		<0.001		<0.001
Policy awareness (as continuous variable)	1.24 (1.17–1.30)	<0.001	1.28 (1.21–1.36)	<0.001	1.18 (1.11–1.25)	<0.001	1.10 (1.03–1.17)	0.003

AOR: adjusted odds ratio; adjusted for district, school type, age, sex, work duration, education level, position in school, training for tobacco control, and smoking status.

## DISCUSSION

The study reported a low tobacco control policy awareness among school personnel in Shanghai, and the awareness was positively related to their anti-tobacco attitudes and behaviors. The findings indicated the importance of improving policy awareness and increasing the involvement of school personnel in tobacco control on campus for adolescent smoking.

The relationship between anti-tobacco policies and smoking-related attitudes and behaviors has been studied in adult smokers before^11,25^. In those studies, the social unacceptability of smoking and smoking cessation were associated with exposure to specific policy measures, such as warning labels on packages and smoking restrictions at work. On campus, teachers in schools with a loosely restrictive tobacco use policy were less concerned about smoking around students and less supportive of anti-tobacco measures than teachers at tobacco-banning schools^26^. However, to our knowledge our study is the first study showing that the more types of anti-tobacco policies school personnel know, their attitudes and behaviors are more supportive towards tobacco control in students. Given the importance of school personnel in students’ behaviors, raising the awareness of anti-smoking policies among this group seems to be of great interest.

In fact, some studies supported the view that the involvement of school personnel in tobacco control campaign on campus can be effective. Increased awareness has been reported to increase compliance with policies and as a result reduce smoking^27^. This could be explained by the KABP model^20^ that is well known in behavioral studies. According to this model, the school personnel who are aware of the anti-tobacco policies may have better knowledge of the provisions (hazard, legal, educations, taxation, counseling/cessation service, etc.) than those who have not heard of these policies. Therefore, first they may hold a positive attitude towards tobacco control, thinking students’ smoking as an unhealthy behavior and likely to step in to help^11^. Second, many policies include detailed action plan so school personnel will be equipped with necessary knowledge and skills to take more effective actions for students^27-29^. Finally, many policies and regulations already include the requirements or suggestions for school personnel in the plan so they will be motivated during the process^30^. Personnel may be more familiar with the earlier regulations (like FCTC, MPOWER and AL in our study), making the association between these regulations and tobacco control behaviors more pronounced in the current study.

One alarming result of the study is that the policy awareness among school personnel is low. Only about half (49.7%) of the surveyed participants were aware of the WHO FCTC, which is similar with the percentage in Nigeria (51.9%)^18^. In Myanmar, 57.3% of the high school students had ever heard about the local tobacco control law: The Control of Smoking and Consumption of Tobacco Products Law^31^. In addition, the awareness of MPOWER and Healthy China 2030 were also low. The low awareness might be due to the fact that the tobacco control effort in China is relatively new. For example, even though the WHO FCTC was signed by China in 2003 and was put into effect in 2006, the first state-level legislation on tobacco control in public places has not been released yet – only a draft put forward in 2014^32^. Fortunately, many local tobacco control regulations (those of Beijing^33^, Guangzhou^34^, Chongqing^35^, etc.) have been enacted in recent years and provided firsthand evidence for the establishment of national law. In addition, some other state-level tobacco control related strategies or regulations have been released in recent years, such as Healthy China 2030 in 2016. In October 2020, The Law of the People’s Republic of China on the Protection of Minors^36^ was revised and was added to the restrictions on the sale of tobacco to minors. Moreover, to cope with the rapid growth of e-cigarette use, Measures for The Management of Electronic Cigarettes^37^ was released by the Tobacco Monopoly Bureau on 11 March 2022.

In our study, many school personnel had not taken many actions regarding students’ smoking. Less than half of the surveyed participants had tried to prevent students from tobacco use, encourage them to quit smoking or participate in relevant educational activities on campus. It is possible that they have a lack of policy access, knowledge about tobacco hazards or little access to tobacco-related materials as suggested by a previous study^10^ and other findings of the current study. As reported before, smoking banning by ‘important people’ (school administrators, teachers) was associated with less tobacco use and more smoking cessation among students^10,11,38^. Given the high prevalence of adolescent smoking in China^4^, increasing involvement of school personnel in students’ tobacco control might be one strategy to help slow the spread of the smoking pandemic on campus.

Regarding the ways of increasing policy awareness and performing more actions, our study showed that receiving training on tobacco use prevention played an important role among school personnel. Specifically, school personnel who had training experience reported to have a higher policy awareness (94.4% vs 85.6%) and perform more actions against students’ smoking compared to those who had no training (41.9% vs 13.1%). However, less than 1 in 5 of the surveyed participants had ever received training related to tobacco control, which may have resulted in a lack of knowledge and techniques to help their students. This is consistent with previous studies in which most personnel had no access to tobacco prevention training (only 8% received training)^10^. The factors for the low training rate might be due to school personnel’s busy schedule or a lack of adequate educational materials. Thus, it would be helpful if tobacco control training can be set as part of their routine work in school, and related educational material offered to teachers, school administrators, and other relevant staff.

Besides training, working duration was also positively associated with policy awareness in our study and other reports^39^. Compared to teachers or other staff, school administrators seemed to have better policy awareness, possibly due to easier access to policies and guidelines as part of their routine work. In addition, personnel in rural areas had even lower policy awareness, and were engaged less in anti-tobacco behaviors/attitudes than those in urban areas in the current study. Although there is no significant difference in policy awareness according to smoking status, current smokers were less likely to support tobacco control measures than never/former smokers. Therefore, training focus could be given more on teachers and personnel with short working duration and smoking currently, especially in rural areas.

### Limitations

Some limitations exist in this study. First, the crosssectional study design cannot determine the direction of causality. Second, our participants were only selected from schools in Shanghai which may not be generalized to other regions. Third, there was no student tobacco use data in our study, thus direct association with students’ tobacco use could not be assessed. In the future, a longitudinal study could be designed in both economically developed and developing areas to explore convincing causality and mechanisms. Besides, future research could include changes in adolescent smoking as part of the results, to analyze the effect of policy awareness better. Intervention studies focusing on improving awareness of anti-tobacco policies and strategies could also be conducted among school personnel.

## CONCLUSIONS

The study calls for school management and local education department to implement projects or activities (like tobacco control training) to improve policy awareness in the school environment as part of school tobacco control strategy. Not only administrators but also teachers and other staff on campus should be taken into consideration especially those who are current smokers and with shorter work durations.

## Data Availability

The data that support the findings of this study are available on reasonable request from the corresponding author.
